# Video consultation in general practice: a scoping review on use, experiences, and clinical decisions

**DOI:** 10.1186/s12913-023-09309-7

**Published:** 2023-03-30

**Authors:** Magnus Repstad Wanderås, Eirik Abildsnes, Elin Thygesen, Santiago Gil Martinez

**Affiliations:** University of Agder, Kristiansand, Norway

**Keywords:** Video consultation, Telehealth, Telemedicine, General practice, Scoping review, Coronavirus disease, Use, Experiences, Clinical decision-making

## Abstract

**Background:**

The coronavirus disease 2019 pandemic forced healthcare workers to use alternative consultation approaches. In general practice, the use of video consultations (VCs) increased manyfold as countries were locked down. This scoping review aimed to summarize scientific knowledge concerning the use of VC in general practice and focused on (1) the utilization of VC in general practice, (2) the experiences of the users of VC in general practice, and (3) how VC affected the clinical decision-making of general practitioners (GPs).

**Methods:**

A scoping review was conducted in accordance with the methodology of Joanna Briggs Institute. Review questions were formulated to match each focus area. A three-step search strategy was employed to search scientific and gray literature sources. MEDLINE, Embase, Scopus, OpenGrey, Google Scholar, and ClinicalTrials.gov were searched from 2010 to March 11^th^, 2021, and the search was re-run on August 18^th^, 2021. The extracted data were deductively coded into pre-defined main themes, whereas subthemes were inductively synthesized. The data within each subtheme were analysed through descriptive content analysis and presented in a narrative synthesis.

**Results:**

Overall, 13 studies were included after screening 3,624 studies. Most patients were satisfied with VCs. VCs were most suitable for simpler issues, often shorter than face-to-face consultations, and were more likely to be used by younger patients. GPs enjoyed the flexibility and shorter duration of VCs; however, they felt an unsatisfactory deterioration in the GP-patient relationship. Despite the loss of clinical examination, diagnostic assessment was mostly successful, with little fear of missing serious illness. Prior clinical experience and a preexisting relationship with the patient were important factors for successful assessment via VC.

**Conclusions:**

Both GPs and patients can be satisfied with VC in general practice in specific contexts, and adequate clinical decision-making is possible. However, disadvantages such as a diminishing GP-patient relationship have been highlighted, and the use of VC in non-pandemic settings is limited. The role of VC in the future of general practice remains unclear, and further research is needed on the long-term adoption of VC in general practice.

**Supplementary Information:**

The online version contains supplementary material available at 10.1186/s12913-023-09309-7.

## Background

The use of video consultations (VC) in primary healthcare has been studied for over 20 years [1]. These studies are often small-scale and focus mainly on the initial adoption of VC in a research context [2]. Few studies have examined the challenges related to the spread and scale-up of VC as a tool for general practitioners (GPs), as most prior studies on VCs have focused on secondary care settings, and the studies focusing on VC in general practice have been confined to considering hypothetical uses, leaving many questions unanswered [3]. Before the coronavirus disease 2019 (COVID-19) pandemic in March 2020, adoption of VC in healthcare, particularly in primary healthcare, was slow [4].

The COVID-19 pandemic forced healthcare providers to adopt alternatives to the traditional face-to-face consultations to prevent viral transmission; therefore, the pandemic provided the opportunity for a “natural experiment” on the widespread adoption of VC [2]. In just one month, from February to March 2020, the use of e-consultations among Norwegian GPs increased by 1000% during the country’s lockdown period. Over 50% of all GP consultations in the first two weeks of lockdown were e-consultations [5], consisting of text-based communication between the patient and doctor, telephone consultations, and VCs. In May 2020, the Norwegian Medical Association reported that the healthcare system conducted approximately 20,000 VCs daily [6]. Several countries have observed similar increases in the use of e-consultations [7–10].

The COVID-19 pandemic (officially declared by the WHO on 12^th^ March 2020 [11]) thus propagated VCs from a place of piloting and limited scale-up to becoming an important consultation modality for general practitioners in several countries to deliver primary health care services. With this uptake in use, VCs were suddenly being put to the test in the real world on a large scale, and updated scientific knowledge on the real-world use of VCs in general practice is likely to have been produced. Going forward, the use of VC in non-pandemic settings will likely be subject to debate as to the extent and settings to which it should be used in general practice. Such a debate would be lacking if recent knowledge is not accounted for. Thus, the main research objective of this scoping review was to summarize the scientific knowledge on the topic of VCs between GP and patient in general practice, in order to inform the discussion around future, non-pandemic use of VC in general practice with an up-to-date knowledge base.

During a preliminary search of MEDLINE and EMBASE (via Ovid), Cochrane Database of Systematic Reviews, SCOPUS, and Joanna Briggs Institute (JBI) Evidence Synthesis, we identified several reviews on telehealth and remote consultations, but only one review (scoping review) by Thiyagarajan et al. [4] focused on video communication for consultation in general practice. Specifically, they studied patients’ and clinicians’ experiences with VCs in primary health care, including empirical research in English language from January 2010 to October 2018.

## Methods

The scoping review was conducted according to the methodological framework described by the JBI [12]. The framework gives an overview of the stages in conducting a scoping review, from defining and aligning the objective(s) and question(s) to presentation of the results, summarizing the evidence, and making conclusions. The JBI recently updated the scoping review framework. As suggested by JBI, the process of evidence selection should be accompanied by a flow diagram showing the process, preferably from The Preferred Reporting Items for Systematic reviews and Meta-Analyses (PRISMA) [14]. The PRISMA flow diagram is found in the Results section.

### Review questions (RQs)

As stated in the background, the main research objective of this scoping review was to summarize the scientific knowledge on the topic of VCs between GP and patient in general practice, to inform the discussion around future, non-pandemic use of VC in general practice with an up-to-date knowledge base. To specify the scope of the review, we chose to focus on three key areas that illustrate important aspects of VC in general practice. These three key areas are (1) utilization of VC in general practice, (2) experiences with VC from both GPs and patients, and (3) clinical decision making with VC. Review questions were formulated on the basis of each of these key areas:

1. How is VC utilized in consultations in general practice?
2. What are the experiences of patients and GPs on the use of VC?
3. What is the impact of VC on the clinical decision-making ability of GPs?

### Inclusion and exclusion criteria

#### Types of participants

We considered studies that involved the use of VC between patients and physicians in primary health care, including GPs. The review excluded studies that focused on VCs in secondary healthcare settings (hospitals, specialist clinics, etc.) and those that examined VCs between more than two participants (e.g., general practitioners, specialists at hospitals, and patients).

#### Concepts

This review included studies that focused on VCs. In cases where broader terms such as “e-consultation” or “telemedicine” were used in the title or abstract, the full text of the study was considered. Studies were subsequently excluded if specific information about VC was not included, and studies were also excluded if the studies examined e-consultation use in general practice without distinguishing the effects of the different modalities of e-consultation, including video consultations, written, asynchronous e-consultations, or telephone consultations. The reason was to exclude studies in which the results could not be attributed to either VC or telephone consultations.

#### Context

This review included studies that focused on the use of VC in primary health care settings, including general practice. It excluded studies that focused on secondary health care settings, as well as mental health services.

### Types of sources of evidence

We included scientific, peer-reviewed studies that met our inclusion criteria, including qualitative, quantitative, and mixed-method studies. The review also included systematic and scoping reviews on the topic of this review. In addition, gray literature (official reports and white papers) was also considered. We included studies published in English and Norwegian languages.

### Search strategy

According to the JBI framework, the search strategy for a scoping review should aim to be as comprehensive as possible, to identify both published and unpublished (gray) primary sources of evidence, as well as reviews [12]. This review developed a search strategy in line with this aim, searching for both published and gray sources of evidence. As suggested by the JBI framework, a three-step search strategy was used. In the first step, an initial limited search was conducted in a selection of appropriate databases (MEDLINE, EMBASE, and Scopus). This limited search was followed by an analysis of relevant studies, including the title, abstract, and the keywords and index terms (standardized topic terms) used to describe the studies. In the second step, another search including all identified keywords and index terms was performed in all the included databases: MEDLINE, Embase, Scopus, OpenGrey, Google Scholar, and ClinicalTrials.gov. In the third step, the reference lists of all the studies included in this review were searched for additional studies. The search was performed from 2010 to March 11^th^, 2021. The full search strategy for all databases can be found in the Appendix.

The search was re-run on August 18^th^, 2021, immediately before the start of evidence synthesis and the writing of this review, to include any new studies published after the first search. This was due to the rapid pace of new literature published on this topic, given that the pandemic was still ongoing.

### Source of evidence screening and selection

Every unique search hit was uploaded to Rayyan [13], an online screening tool. In Rayyan, studies were screened and selected based on titles and abstracts. The first author (MRW) screened all the studies, whereas the co-authors (SGM, ET, and EA) screened one-third each, securing a double screen for every study. MRW held individual online meetings with each of the three co-authors after two weeks of screening, where any misunderstanding during the screening process was resolved.

The full text of studies selected during the title-abstract screening were then screened to assess if they met the inclusion criteria. Again, MRW screened all selected studies, whereas SGM, ET, and EA screened one-third each, securing a double screen for every study. The studies that were selected for inclusion in the review were independently selected by two authors. Disagreements were resolved through online meetings. Eligible studies were included after full-text screening. Uncertainty pertaining the terms or concepts used in the studies was resolved through e-mail correspondence with the corresponding authors of the included studies. An example of such uncertainty was the term “digital consultations” in the study by Fernemark et al., which the corresponding author confirmed was a synonym for VC.

### Data extraction

Data were extracted from the included studies using a charting table formatted in a Microsoft excel spreadsheet. The extracted data included specific details regarding the population, concept, context, study methods, and findings relevant to the objectives and review questions of this study. From the two included reviews, textual data was extracted from both the results and the discussion sections of the studies. Two authors independently extracted data from each included study. Any disagreements between the authors were resolved through a discussion with a third author. The data extraction form is provided in the Appendix.

In addition to transferring the data extracted to a data extraction form, the included studies were uploaded into NVivo (V. 12. QSR International, USA, Burlington, Massachusetts), a computer-assisted qualitative data analysis software. In NVivo, the uploaded data were deductively coded into pre-defined main themes that matched the review questions and, thus, focused on results relating to the use of VC, the experiences of VC users, and clinical decision-making using VC. Within each theme, several subthemes were synthesized in an inductive manner, through capturing emerging patterns in the extracted data.

### Analysis and Presentation of results

The data within each inductively synthesized subtheme were analysed through descriptive content analysis [12]. The results are presented in a narrative synthesis, as this allows for summarising the findings from both the qualitative and the quantitative studies. A tabular summary of the included studies, which shows the author, year, title, study method, and study objective as well as the study country, setting (rural or urban), number of GPs and patients, and whether the study focused on VC alone or VC together with other e-consultation modalities, is also presented.

## Results

### Search results

**Fig. 1:**
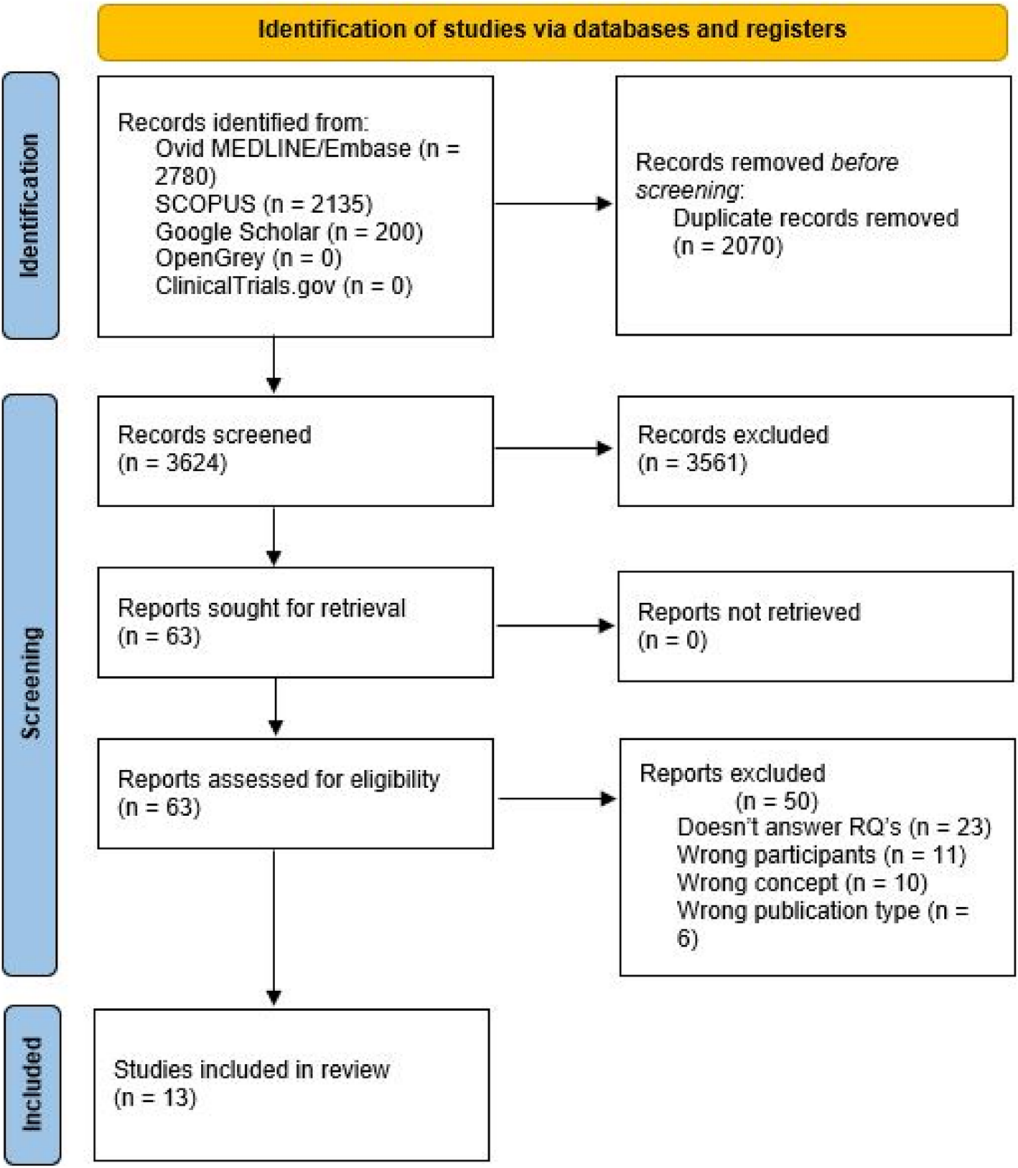
PRISMA selection process [14]

Thirteen studies were included in the present review. The PRISMA flow diagram (Fig. 1) outlines the search and selection process. A tabular summary of the included studies is presented in Table 1.

**Table 1.**
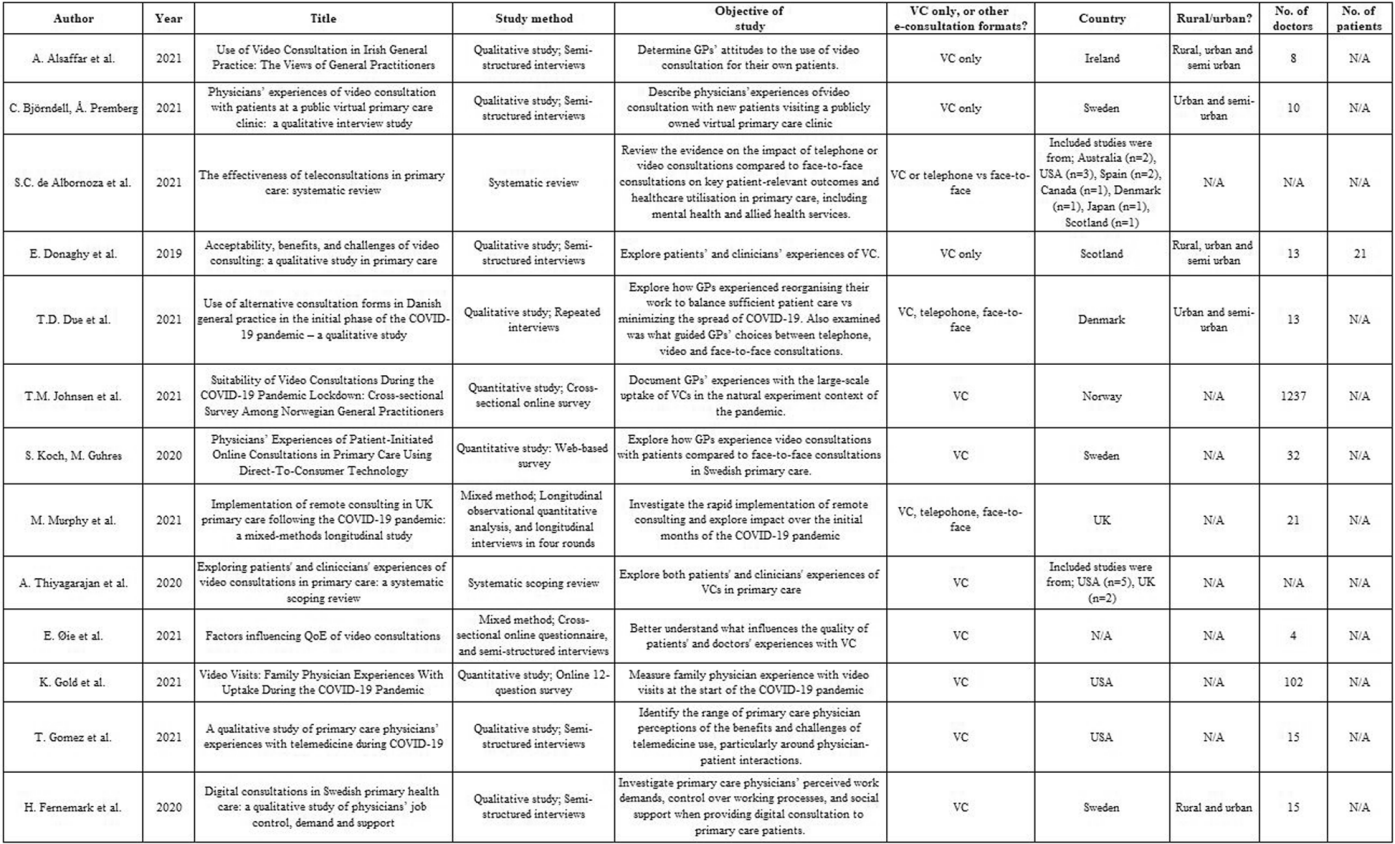
Tabular summary of included studies

### Utilization

All 13 studies reported results regarding RQ1, which included information on how VCs are utilized in general practice. The results were divided into three subthemes: consultation content, consultation metrics, and user demographics.

#### Consultation content

Several studies highlighted suitable ways to utilize VCs. Follow-up consultations and review appointments were also frequently reported. In one survey study, 55% of approximately 3,500 consultations were follow-up consultations, and in over 60% of these, VCs were judged to be better or as good as face-to-face consultations [15]. VC was deemed suitable for conveying laboratory results, adjusting medications, and managing chronic diseases [4, 16, 17]. VC was also deemed suitable for managing mental health issues [15–19]. Furthermore, VCs were deemed suitable for simpler and more straightforward issues, including administrative tasks, such as sick leave or certification [15, 16, 18, 20, 21].

#### Duration and number of complaints

Several studies reported findings concerning the duration of VCs and the number of patient complaints during a VC. One study reported that GPs felt VCs were time-neutral [3]. However, a second study found VCs to be an average of 8 min shorter than the average time of face-to-face consultations [22], and a third study found the average time duration to be less than half that of face-to-face consultations, from over 20 min to under 10 min [23]. Regarding the number of patient complaints, one study stated that patients had fewer complaints during VCs compared to face-to-face consultations, which might be due to the higher availability of virtual appointments [21]. One study found that on average, 1.9 complaints were reported during each VC [15], whereas another found an average of 1.5 complaints [3].

#### User demographics

Three studies reported the demographic characteristics of VC users. One study found that VC was significantly more likely to be used by younger patients and GPs, with no differences between sexes [19]. They found no differences between rural and urban settings or socioeconomic gradients [19]. Another study found that VC use among GPs varied from almost exclusively VCs to as few VCs as possible, and this variation was not linked to practice type, sex, or seniority [20]. The last study emphasized the usefulness of VCs in connecting with patients in nursing homes [24].

### Experiences

All 13 studies reported results regarding RQ2 including patients’ and GPs’ experiences with using VC. The results were divided into four subthemes: patient experiences, GP experiences, the GP-patient relationship, and experiences with VC technology.

#### Patient experiences

Convenience was a recurring positive factor among patients, with reduced travel times, no waiting in the GP’s waiting room, and improved access to GPs [3, 4, 18, 25]. Patients felt that VC was “less stressful” than visiting the GP office [3]. In one study, approximately 94–99% of patients were “very satisfied” with VC, with 95% stating that they would want to use VC again [4]. One study found that visual cues with VC was an advantage when compared to telephone consultations [3], whereas another study mentioned visual cues as an aspect that was missing in VC compared with face-to-face consultations [18]. One randomized control trial found that patients preferred face-to-face consultations over VCs and that this preference was more apparent in patients with chronic diseases [4].

Several studies reported patient experiences as observed by GPs. In several studies, GPs had the impression that patients were generally very happy with VC as a consultation platform [4, 15, 25]. Owing to the flexible nature of VC, patients missed fewer appointments and experienced less inconvenience with accessing the GP’s office [17]. However, one study reported that patients might have had very high expectations of VCs and that they had a poor understanding of the complaints that were appropriate for video appointments [23].

#### GP experiences

Among 100 GPs, over 90% were either very satisfied or somewhat satisfied with VCs [22]. One advantage of VCs that was frequently mentioned was the flexibility of GPs’ work situations, with an increased flexibility of working from home [21, 23, 25]. Another advantage was the possibility of seeing patients’ homes and their environment, together with interacting with family members when necessary [17]. In some studies, GPs reported that VCs felt more focused and straightforward and they enjoyed these shorter consultations [17, 20]. Participants in one study reported that VCs compared with face-to-face consultations led to a lower workload, whereas another study found no clear difference in workload between face-to-face consultations and VCs [23, 25].

Regarding the disadvantages, one study reported on how GPs could be frustrated over their inability to address the patients concerns via VC [21]. Another study highlighted the low adoption of VCs despite positive comments from patients and employees, which might be explained by the relatively limited usefulness of VCs compared with telephone or face-to-face consultations in most situations [24]. Juggling between face-to-face consultations and VCs was identified as a major stress-inducing element in the daily work of GPs [17]. Several GPs were worried that VC might reduce the value of general practice and that adoption of VC could, in the worst case, compromise the current role of general practice [16]. Others feared that increased use of VCs might create or worsen health access inequality, where older people and disadvantaged patients be the most affected [4, 24, 25].

#### The GP-patient relationship

Several studies discussed the effect of VC on patient-GP interactions and relationships. Several GPs feared that a lack of face-to-face encounters would have a negative effect on the GP-patient relationship [16–18]. Compared to face-to-face consultations, GPs mentioned difficulties with nonverbal communication and missing social cues with VCs [17, 20]. One study highlighted the need for physical touch and examination as a means of performing “expected rituals,” which are important in the GP-patient relationship [17]. The digital divide can lead to a more anonymous form of communication with patients saying inappropriate things [25] and withholding personal information [17]. However, many GPs agreed that VC is superior to telephone consultations regarding communication and rapport [3, 19, 20].

#### Experiences with VC technology

Experiences with the technological side of VC varies. Several studies reported difficulties with VC technology and how unstable internet connections hampered consultation quality [3, 4, 18, 22]. Other studies found that participants experienced good technological quality [23, 25], with one study reporting that GPs were satisfied with the technology in almost 90% of consultations [15]. One study highlighted that older patients required considerable assistance to participate in VCs [17].

### Clinical decision-making

Twelve of the 13 included studies reported results related to RQ3, which included the impact of VC on the clinical decision-making ability of GPs. The results were divided into three subthemes: assessment via VC, effect on measures, and patient safety.

#### Assessment via VC

One study summarized that more than 88% of over 700 GPs felt that clinical decision-making was successfully achieved through VC [4]. In a survey of almost 3,500 VCs performed during the initial phase of the pandemic, over 50% of GPs felt that VC compared with face-to-face consultations was as good or better at assessing the severity of the main reason for contact [15]. Gold et al. reported a similar number, with only 41% of GPs stating that face-to-face consultation would have been a better consultation approach [22].

Allergies were commonly reported as a disorder that is suitable for VCs [25]. VC was also useful in cases where dynamic assessment was necessary, such as gait and respiratory monitoring, as well as useful in assessing children [20, 24]. In one study, skin diseases and mild infections were highlighted as cases suitable for VC [21], although another study reported that assessing skin diseases using this approach was challenging, mostly due to the inability to feel the rash [17]. Musculoskeletal disorders were highlighted by GPs as disorders that could be managed via VC, whereas others felt VC was not unsuitable for such disorders [20]

Lack of physical examination meant that potentially serious conditions, such as chest pain, abdominal pain, or neurological symptoms, were difficult to assess through VC [15, 17, 18]. Several studies reported this lack of physical examination as a clear limitation of VC [16, 17, 24]. Among the previously mentioned 3500 VCs, GPs reported that lack of examination was a “major loss” in 25% of VCs, and in 40% of VCs, GPs reported “no loss” regarding the inability to examine the patient [15]. One study reported that GPs sometimes guided patients through self-examination to minimize the loss of physical examination [21].

Two studies reported on the insufficiency of VCs in most cases and the possibility of missing important information through VCs [20, 22]. However, Due et al. [20] highlighted that diagnostic uncertainty was not a prominent issue, with the possibility of scheduling follow-up appointments as a form of safety net. Johnsen et al. [15] found that GPs feared missing potentially serious illnesses in only 15% of consultations using VCs*.* A preexisting relationship with the patient [15, 18, 20] and having clinical experience in traditional general practice [21, 23] were highlighted as important factors that increased the suitability of VCs.

#### Effect on measures

The nature of VCs can result in GPs taking alternative measures compared with that observed during face-to-face consultations. For instance, patients with suspected asthma could, without spirometry, be started on a trial-and-error-type treatment regime [19]. In addition, some studies reported that not seeing and examining patients physically led to a more liberal pharmaceutical approach, especially over-prescription of antibiotics [17, 20]. However, some GPs felt it was easier to refuse unwarranted patient requests via VCs than during face-to-face consultations [17]. One study found that a slight majority of GPs were uncomfortable with referring patients to secondary care facilities through VCs [23].

#### Patient safety

One study argued that missing guidelines on the use of VC might pose a threat to patient safety, highlighting the need for clearer guidelines and boundaries that specify the conditions that VC is suitable for [16]. This notion was mirrored by another study that reported how patient safety might be negatively affected through VCs for conditions that were not suitable for remote assessment [25]. Two studies also highlighted how VCs performed at the same health center with a unified documentation system could improve patient safety [21, 25].

## Discussion

The main objective of this scoping review was to summarize knowledge on the utilization of VC in general practice, patients’ and GPs’ experiences with VC, and how clinical decision-making among GPs is achieved through VCs. There was a high degree of agreement across the included studies regarding utilization of VCs. Regarding experiences from GPs and patients, and clinical decision making with VC, the results spanned across a range of both positive and negative outcomes.

Many of the findings in this review regarding experiences with VC among patients and GPs matched the findings of the one previously published scoping review on the topic, by Thiyagarajan et al. [4]. Compared to the previous review, this scoping review differs in three important ways. Firstly, this review examined two other aspects of VC in general practice, namely utilization and clinical decision making. Secondly, several of the included studies in this review dealt with peripandemic settings, thus capturing findings from organic, unplanned use of VCs. A third important difference between the two reviews is how this review looks closer at the effect of VC on the GP-patient relationship.

In the results sections concerning experiences with use of VC and clinical decision making with VC, there is an abundance of contradictions between studies. For instance, one study found VCs leading to lower workloads for physicians, whereas another found no difference in workload. Likewise, there were conflicting results regarding the usefulness of VCs in assessing skin diseases and musculoskeletal disorders. The presence of such contradictions might point to a broader issue – namely the appearance that the topic of VC in general practice is a fragmented field of research. However, instead of thinking of this field as fragmented, one should recognize possible underlying causes that make this topic difficult to compare. First, studies on this topic deal with general practice patients – that is, patients that cover the whole demographic spectrum, with a wide span of diseases that range from trivial to incurable and chronic. Second, studies have varying comparators for VCs when evaluating effects – mainly face-to-face consultation and telephone consultations. Third, studies stem from countries with different economies and diverse health care systems, where the role of GPs can differ markedly. Going forward, new studies should aim to reduce fragmentation to ensure that this field of research may progress towards a more comprehensive and cohesive body of knowledge. One way to achieve this, is to standardize the comparator – preferably face-to-face consultations in our opinion. Another possibility is to design prospective studies evaluating effects of VCs versus face-to-face consultations for select patient groups that are more easily compared across countries – for instance, follow-up of diabetes, chronic obstructive pulmonary disease (COPD) or fibromyalgia.

Despite the disadvantages with VCs described in this review, the results seem to show that VC has the potential to become a part of routine general practice in a post-pandemic setting, given the high satisfaction with the consultation format, and that safe clinical decision-making can be achieved when VC is used for suitable, non-acute illness. One could therefore expect the increased use of VC to be maintained after the pandemic. However, numbers from the Norwegian Directorate for E-health showed that in the first months of the pandemic, from March to June 2020, the use of VCs in Norwegian general practice fell by over two-thirds, from a weekly average of approximately 6,200 VCs daily to below 2,000 VCs daily [26]. This illustrates that even though GPs gained experience with the consultation format, they still chose other consultation modalities as societies gradually eased the COVID lockdown restrictions. Some of the explanations for this decline in VC use may be that Norwegian GPs received the same amount of reimbursement from a telephone consultation as from a VC, implying that GPs lacked economic incentives regarding the use of VCs. Other reasons may be a lack of organizational capacity for change in a hectic workday, or that GPs found the technology offered limited usefulness.

The difficulties of establishing VC in general practice have also been demonstrated in the UK, where VC use was, in most cases, either never adopted or was used for only a short term [27]. In December 2021, video and e-consultation made up less than 0.5% of the total number of GP consultations in England, and although GPs reported some advantages of VCs, such as out-of-hours and nursing home consultations, in most cases, the perceived advantage of VCs was minimal. GPs found that most issues could either be addressed over the telephone or required face-to-face consultation. Further research needs to be conducted to understand more of the decline of VC in general practice.

Several included studies in this review stated that VC negatively affects the GP-patient relationship [16–18]. This may pose a dilemma in the future with the increased use of VC in general practice. A recent Norwegian study found a significant association between the length of the GP-patient relationship and the use of out-of-hours services, hospital admissions, and even mortality [28]. This shows the importance of the GP-patient relationship, an element that gives general practice its unique value, which should be maintained in this era where the use of digital health technology is increasing. These technologies have been used for a short period; therefore, researchers have not yet assessed whether the same kind of GP-patient relationship can be achieved with extensive use of digital communications. However, among the included studies, three highlighted that VCs were superior to telephone consultations in building such a relationship [3, 19, 20]. This might indicate that in a possible future healthcare system with increased digital communication, VC may be an appropriate choice to maintain the strength of GP-patient relationship.

There is also a need to assess whether VC can be a solution in addressing challenges related to the demographics of an ageing and multimorbid population, with increased healthcare utilization and more complex psychosocial needs [29]. One study in this review showed how VC is more likely to be used by younger patients, while another study found older patients requiring assistance to participate in VCs. The results also show how patients with chronic diseases prefer face-to-face consultations over VC and highlight a fear of how increased use of VCs may be accompanied by health access inequalities where older and disadvantaged patients might face challenges. Digital health disparities – “*inequalities that may be widened when technologies are required for accessing and receiving care*” – are likely to escalate as societies become increasingly digitized [30]. Considering this, it seems that VC as of today may not be a major contributor to addressing these demographical challenges.

What is the future of VC in general practice? Research analyzing VCs during COVID-19 from a practice theory perspective, stated in its conclusion that “*the future of video consulting is inherently unpredictable*” [31]. This scoping review has shown that VC, in light of utilization, experiences and clinical decision making, can be a component of general practice. However, the declining use of VC may show that there is another perspective that is equally, if not more, important in gauging how VC fits in future general practice – namely, the preferences of GPs regarding general practice. Face-to-face meetings, strong GP-patient relationships, and continuity of care are fundamental pillars of strong general practice, and the introduction of VC and other forms of digital communications might be perceived as a challenge to these pillars.

A fundamental issue facing health care systems in many countries is how digital health technologies can be incorporated to increase efficiency while at the same time delivering quality healthcare services. In general practice, this issue may force the rethinking of how general practice is organized. Until now, the face-to-face consultation has been considered the gold standard for providing best possible healthcare. Can digital technologies raise the bar for the gold standard consultation? Or even create a new gold standard? The studies in this review have evaluated “VC version 1.0” – that is, a digital video dialogue between patient and GP. How will “VC version 2.0” look? One possibility is *augmented* VCs, which in addition to the video dialogue, allows for engaging in virtual physical examinations with support from the staff, capturing vital signs and examinations using stethoscope and otoscope. Experiences with augmented VCs between GPs and patients in care homes, as well as care home staff, showed that it promoted person-centered care in meeting the needs of older adults. [32].

Perhaps technologies like VC and augmented VC are necessary for general practice to adapt to increased demands. However, as work continues with digitizing the GP consultation, one should keep in mind the potential for losing some of the value found in the face-to-face consultation – the undefined and unspoken aspects, the pillars of general practice. As research progresses in the field of digitizing the GP consultation, efforts should be made to maintain and respect the unique nature of general practice.

### Future research

Future research on this topic should aim to design studies that facilitate comparison of results and effects, to build a comprehensive and cohesive body of knowledge. It is important to better understand why GPs stop using, or reduce the use of, VC. Research should also aim to evaluate how GPs themselves envision a future general practice, and to what extent VCs should be incorporated. The studies in this review do not give a unison knowledge base that can be used to develop guidelines or frameworks for the most appropriate use of VCs in general practice, and this should be a focus area in future research on this topic. To better inform this, patient preferences should also be better examined.

As the use of digital consultations progresses, research on how these forms of communication impact the GP-patient relationship could be essential to further strengthen the understanding of the role of digital consultations in general practice. Future research should also be planned with longitudinal designs, comparing the long-term quality of general practice healthcare delivered over digital platforms to healthcare delivered in traditional face-to-face consultations, to capture potential downsides in healthcare quality with a gradual increase of digitalization in general practice.

### Strengths and limitations

The included studies originated from several countries, each with its own primary healthcare structure. Although many of these countries are neighboring countries with similarities in healthcare systems, the results from these studies are still affected by between-country differences. This can make it difficult to compare experiences with digital technology among the studies because of the different settings.

In the inclusion/exclusion criteria, we excluded studies that examined e-consultation use in general practice without distinguishing the effects of the different modalities of e-consultation, including video consultations, written, asynchronous e-consultations, or telephone consultations. Although the exclusion was an important to limit confounders regarding experiences with different e-consultation modalities, some relevant data may have been missed.

## Conclusion

This scoping review showed that both GPs and patients can be satisfied with VC in general practice in specific contexts of use, and that adequate clinical decision-making is possible with VC. However, disadvantages such as a diminishing GP-patient relationship have been highlighted, and the use of VC in non-pandemic settings is limited according to usage numbers from Norway and the UK. Further research on VC in general practice should aim to develop a comprehensive and cohesive body of knowledge on the adoption of VC in general practice, as well as consequences of digitizing the GP consultation.

## Supplementary Information

**Additional file 1.**

## Data Availability

The data that support the findings of this study are available from The Norwegian Directorate for E-health, but restrictions apply to the availability of these data, which were used under license for the current study, and so are not publicly available. Data are however available from the authors upon reasonable request and with permission of The Norwegian Directorate for E-health.

## Abbreviations

VC: Video consultations
GP: General practitioner
UK: United Kingdom
COVID-19: Coronavirus disease 2019
JBI: Joanna Briggs Institute
RQ: Review question
PRISMA: The Preferred Reporting Items for Systematic reviews and Meta-Analyses
