# Supplementary material for: Video consultation in general practice: a scoping review on use, experiences, and clinical decisions
Source: BMC Health Serv Res. 2023 Mar 30;23:316. doi: 10.1186/s12913-023-09309-7 (PMC10063329; doi:10.1186/s12913-023-09309-7)
Supplement: Supplementary file 1 — Additional file 1. [file 12913_2023_9309_MOESM1_ESM.docx]

**Appendices**

**Appendix 1: Search strategies for all databases**

**Database: Embase, Ovid MEDLINE(R) ALL**

Search Strategy:

1 general practi*.ti,ab,hw,kf,kw.

2 (primary adj3 (health* or care)).ti,ab,kf,kw.

3 Primary Health Care/

4 ((general* or family or primary) adj3 (doctor* or practi* or physician* or medicine)).ti,ab,kf,kw.

5 (video* adj4 (consult* or care or visit* or appoint* or call* or link* or conf* or teleconf*)).ti,ab,kf,kw.

6 telemedicine/ and consultation/

7 telehealth/ and consultation/

8 videoconferen*.kw,kf.

9 remote consultation/

10 teleconsultation/

11 ((digital* or home or online or virtual*) adj3 consultation*).ti,ab,kw,kf.

12 or/1-4

13 or/5-11

14 12 and 13

15 limit 14 to yr="2010 -Current"

**Database: SCOPUS**

Search strategy:

TITLE-ABS-KEY(("general practi*")

OR (primary W/3 (health* OR care))

OR ((general* OR family OR primary) W/3 (doctor* OR practi* OR physician* or medicine)))

AND

TITLE-ABS-KEY((video* W/4 (consult* OR care OR visit* OR appoint* OR call* OR conf* OR teleconf*))

OR ((digital* OR home OR online OR virtual* OR remote) W/3 consultation*)

OR teleconsultation*)

AND PUBYEAR aft 2009

**Database: Google Scholar**

Search strategy:

"video consultation|consultations|consulting|care|visit" physician|physician|doctor|doctors|primary

**Database: ClinicalTrials.gov**

Search strategy:

Search string “video consultation, general practice” in the box “other terms”.

We chose only “completed studies”.

**Database: OpenGrey**

Search strategy:

(("general practi*")

OR (primary NEAR/3 (health* OR care))

OR ((general* OR family OR primary) NEAR/3 (doctor* OR practi* OR physician* or medicine)))

AND ((video* NEAR/4 (consult* OR care OR visit* OR appoint* OR call* OR conf* OR teleconf*))

OR ((digital* OR home OR online OR virtual* OR remote) NEAR/3 consultation*) OR

teleconsultation*)


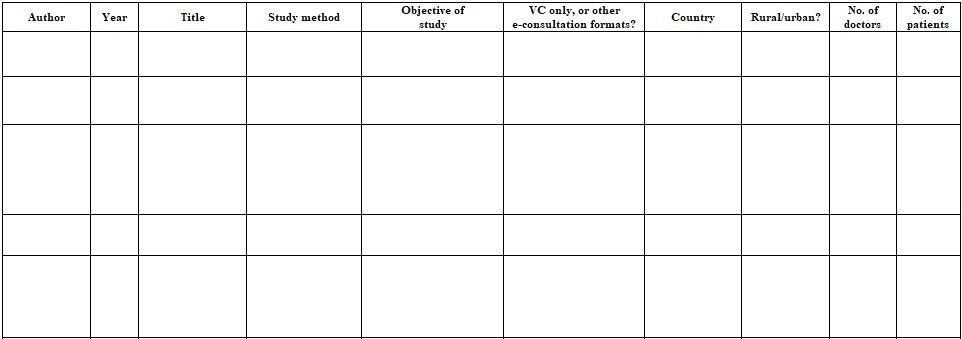
**Appendix 2: Data extraction form**
